# Combined Exercise Training and Nutritional Interventions or Pharmacological Treatments to Improve Exercise Capacity and Body Composition in Chronic Obstructive Pulmonary Disease: A Narrative Review

**DOI:** 10.3390/nu15245136

**Published:** 2023-12-18

**Authors:** Bente Brauwers, Felipe V. C. Machado, Rosanne J. H. C. G. Beijers, Martijn A. Spruit, Frits M. E. Franssen

**Affiliations:** 1 Department of Research and Development, Ciro, Centre of Expertise for Chronic Organ Failure, 6085 NM Horn, The Netherlands; martijnspruit@ciro-horn.nl (M.A.S.); frits.franssen@ciro-horn.nl (F.M.E.F.); 2 NUTRIM School of Nutrition and Translational Research in Metabolism, Faculty of Health, Medicine, Life Sciences, Maastricht University, 6229 HX Maastricht, The Netherlands; 3 BIOMED (Biomedical Research Institute), REVAL (Rehabilitation Research Centre), Hasselt University, 3590 Hasselt, Belgium; felipe.machado@uhasselt.be; 4 Department of Respiratory Medicine, NUTRIM Research Institute of Nutrition and Translational Research in Metabolism, Maastricht University Medical Center+, 6200 MD Maastricht, The Netherlands; r.beijers@maastrichtuniversity.nl

**Keywords:** COPD, nutrition, pharmacological treatments, nutritional supplements, exercise capacity, muscle perseverance, weight loss

## Abstract

Chronic obstructive pulmonary disease (COPD) is a chronic respiratory disease that is associated with significant morbidity, mortality, and healthcare costs. The burden of respiratory symptoms and airflow limitation can translate to reduced physical activity, in turn contributing to poor exercise capacity, muscle dysfunction, and body composition abnormalities. These extrapulmonary features of the disease are targeted during pulmonary rehabilitation, which provides patients with tailored therapies to improve the physical and emotional status. Patients with COPD can be divided into metabolic phenotypes, including cachectic, sarcopenic, normal weight, obese, and sarcopenic with hidden obesity. To date, there have been many studies performed investigating the individual effects of exercise training programs as well as nutritional and pharmacological treatments to improve exercise capacity and body composition in patients with COPD. However, little research is available investigating the combined effect of exercise training with nutritional or pharmacological treatments on these outcomes. Therefore, this review focuses on exploring the potential additional beneficial effects of combinations of exercise training and nutritional or pharmacological treatments to target exercise capacity and body composition in patients with COPD with different metabolic phenotypes.

## 1. Introduction

Chronic obstructive pulmonary disease (COPD) is a chronic respiratory disorder that is associated with significant morbidity, mortality, and healthcare costs [1,2,3]. It is the third leading cause of death worldwide and caused 3.23 million deaths in 2019 [1,4,5]. COPD is characterized by persistent airflow limitation and respiratory symptoms such as dyspnea, sputum production, and cough that are caused by an individually varying combination of chronic bronchitis and emphysema [1,4,6,7]. Moreover, patients may suffer from severe fatigue and comorbid chronic conditions, including osteoporosis and cardiovascular disease [8,9,10]. The burden of these symptoms can translate to reduced physical activity. Physical inactivity can result in structural and functional changes in the lower-limb muscles, such as muscle atrophy, shift in fiber type distribution, reduced capillary density, and reduced muscle oxidative capacity, which in turn contribute to poor exercise capacity in patients with COPD [11]. Subsequently, patients generally have a limited ability to perform tasks in daily life and their health-related quality of life (HRQL) is often decreased [12,13].

While inhaler therapies targeting the lungs remain the core treatment of COPD, it is acknowledged that extrapulmonary features of the disease such as exercise intolerance, muscle dysfunction and body composition abnormalities also require treatment. Pulmonary rehabilitation (PR) is an evidence-based intervention that offers the opportunity to target these treatable traits [14]. PR focuses on providing patient tailored therapies to improve both physical and psychological status of patients with COPD through education, exercise training, nutrition, and behavior modifications [15,16,17,18]. These patient-tailored therapies offered during PR can be strategically combined to address the unique characteristics of individuals within specific phenotypic characteristics. According to the latest European Respiratory Society statement on nutritional assessment and therapy in COPD, patients with COPD can be divided into metabolic phenotypes based on body mass index (BMI) and fat-free mass index (FFMI). These metabolic phenotypes include cachectic, sarcopenic, normal weight, obesity, and sarcopenic with hidden obesity [19].

Weight loss, underweight, and low fat-free mass (FFM) are associated with increased mortality. In contrast, obesity is associated with decreased mortality in severe COPD, but with potentially increased cardiovascular risk [15,20,21,22,23]. In obese individuals, low muscle mass is often hidden through increased fat mass, and this can also be the case in patients with a normal weight [24]. Physical inactivity, systemic inflammation, smoking, and poor dietary quality can impact muscle mass, function, and exercise capacity [15].

Exercise training in patients with COPD can partly counteract the extrapulmonary features of the disease by improving exercise capacity, upper and lower-limb muscle strength, dyspnea, fatigue, and HRQL [25,26,27,28,29]. Besides exercise training, nutritional interventions may contribute to improving body composition, exercise capacity, muscle function, and HRQL [30,31]. Nutritional adjustments that are made during PR aim mainly to support exercise training. In addition, they target body composition by either enhancing weight loss and maintaining muscle mass in obese patients, or attempt to promote weight gain, and specifically increase muscle mass in cachectic or sarcopenic patients [32].

According to a recent systematic review, exercise training or nutritional supplementation in isolation offer limited improvements in measures of FFM, a surrogate for muscle mass among patients with COPD [33]. Consequently, the concurrent implementation of exercise and nutritional interventions emerges as a promising therapeutic approach, with preliminary investigations demonstrating its potential efficacy [33]. For example, in malnourished patients with COPD defined as BMI < 19 kg/m^2^, nutritional supplementation in combination with low-intensity exercise increased body weight, FFM, quadricep muscle force, and walking distance, and decreased dyspnea, when compared to a control group [34]. A more recent study combined nutritional supplementation with high-intensity exercise training in patients with normal BMI but low muscle mass, and showed additional improvements in nutritional status, inspiratory muscle strength, and physical activity when compared with placebo [35]. Combining exercise and nutritional interventions may also enhance treatment efficacy for obese patients with COPD [36].

This narrative review:

- Summarizes the main skeletal muscle adaptations in response to exercise training in healthy individuals and patients with COPD;
- Discusses the potential additional beneficial effects of nutrition when combined with exercise training to target exercise capacity;
- Describes the potential additional effects of nutrition or pharmacological treatments when combined with exercise training to target muscle perseverance and/or growth;
- Presents the potential additional effects of nutrition or pharmacological treatments when combined with exercise training to target weight/fat loss;
- Explores novel compounds that have shown ergogenic potential in healthy populations with limited evidence in patients with COPD.

## 2. Methods

A PubMed search was performed in September and October 2023, and an updated search was performed in November and December 2023. Search terms that were used were ‘healthy individuals’, ‘athletes’, ‘elderly’, ‘COPD’, ‘pulmonary rehabilitation’, ‘metabolic phenotypes’, ‘exercise performance’ or ‘exercise capacity’, ‘exercise training’, ‘resistance training’, ‘endurance training’, ‘muscle perseverance’ or ‘muscle maintenance’, and ‘weight loss’. These terms were then appropriately combined with nutritional interventions or pharmacological treatments: ‘nutritional support’, ‘PUFAs’, ‘caffeine’, ‘creatine’, ‘nitrate’ or ‘beetroot juice’, ‘beta-alanine’, ‘vitamin B12’, ‘vitamin D’, ‘l-carnitine’, ‘protein’, ‘essential amino acids’, ’leucine’, ‘anabolic-androgenic steroids’, ‘activin type II receptor blockade’, ‘ghrelin’, ‘hypocaloric diets’, ‘glucagon-like peptide-1 receptor agonists’, ‘sodium bicarbonate’, ‘vitamin C’, ‘vitamin E’, ‘magnesium’, ‘(-)-epicatechin’, ‘nicotinamide riboside’, ‘β-hydroxy β-methylbutyrate’. There were no additional search criteria applied. In case meta-analysis or systematic reviews had been published on the topic, the most recent article was selected and the articles included in these reviews were thoroughly checked. In case more recent articles were published that were not included in these reviews, these articles were also selected and discussed in this review. If no meta-analyses were available, all available randomized controlled trials (RCTs) were selected and summarized. In the current narrative review, the GRADE framework was not applied, and therefore, no clinical recommendations will be provided.

### 2.1. Different Types of Exercise Training 

Exercise is a type of physical activity that consists of planned, structured, and repetitive bodily movement performed to improve and/or maintain one or more components of physical fitness [37]. Exercise can be modified by changing the modality (e.g., endurance vs. resistance), frequency, duration, intensity of exercise bouts, and type of training (e.g., walking, cycling, etc.) [38]. These modifications can lead to differences in metabolic and molecular responses to exercise [38].

Frequent exercise training is a potent stimulus for physiological and functional adaptations in skeletal muscle [38,39]. Training-induced adaptations in response to contractile activity in the muscle include changes in muscle protein synthesis (MPS), muscle protein breakdown (MPB), mitochondrial function, intracellular signaling, and metabolic regulation, as well as transcriptional responses, and are different among training modalities [38,40].

Because of these diverse adaptations to different types of exercise, a short overview of the different training modalities, along with their underlying mechanisms and resultant adaptations, is provided below.

### 2.2. Resistance Training 

Resistance training, also known as strength training, aims to improve muscle mass and strength [41]. Resistance training is performed against an external resistance by using free weights, machine weights, resistance bands, or body weight to increase muscle mass [41,42]. Resistance training stimulates remodeling of the skeletal muscle by positively influencing both muscle MPS and muscle protein MPB [43,44]. To obtain improvements in MPS, muscle mass and strength, the principle of overload must be applied by manipulating the number of repetitions, intensity, or recovery periods between sets, and the frequency of training sessions [44,45,46].

Short-term adaptations to several weeks of resistance training on whole-muscle level are noticeable in increases in strength and stimulation of MPS, but without increases in muscle size. Long-term adaptations to several months of resistance training on muscular level are muscle hypertrophy [41,47], increases in muscular strength [44], and neural adaptations [44]. Resistance exercise also increases blood flow to the working muscles, thereby improving the delivery and absorption of hormones and nutrients to the muscle, which generates an anabolic environment [44]. On cellular level, after exercise, mechanical and chemical factors interact with hormonal and nutrient signals to regulate enzymes and mediate MPS through the transcription and translation of proteins [44]. MPS rates generally rise between 45 and 150 min after exercise, and may be sustained for up to 4 h in a fasted state, and up to and beyond 24 h in fed state with increased amino acid (AA) availability [45,48]. These changes in MPS are predominantly regulated by changes in mTOR signaling and AMPK signaling [49,50].

### 2.3. mTOR Signaling 

The protein kinase mechanistic Target of Rapamycin Complex 1 (mTORC1) plays a key role in increasing MPS and acts through the Akt-mTOR1-p70S6K pathway [39,51]. This pathway is activated by either mechano-sensation, increased intracellular AA, or in particular, essential amino acid (EAA) concentrations [52,53,54,55], insulin, or insulin-like growth factor 1 (IGF-1) [56]. Specifically, mTORC1 regulates MPS by phosphorylating downstream signaling factors such as p70S6k and 4E-BP1, which are the major regulators of the translation initiation of protein synthesis [43,56]. After acute resistance training, myofibrillar protein synthesis increases in trained and untrained individuals, while mitochondrial protein synthesis only increases in untrained individuals [50]. In addition, mRNA expression of several skeletal muscle AA transporters is upregulated following resistance training and/or EAA ingestion, playing a large role in transporting AA across the membrane and targeting downstream factors that regulate MPS [57,58,59].

### 2.4. AMPK Signaling 

Intracellular energy status is sensed by training, adenosine monophosphate-activated protein kinase (AMPK). Low ATP:ADP ratio activates AMPK which reduces mTORC1 activation through a series of signaling actions. This way, AMPK signaling reduces MPS when there is a low energy status [49]. Previous research has shown that after acute resistance training, when energy levels are diminished, AMPK phosphorylation increases, but returns to baseline values 4 h after training [50].

### 2.5. Resistance Training in Patients with COPD 

Previous research demonstrated that resistance training is beneficial in normal weight, overweight, and obese patients with COPD, as it has shown to improve muscular strength [25,27,60,61,62,63,64,65,66,67,68], leg lean mass [66], exercise capacity [25,27,60,61,62,63,64,66,67,68,69], dyspnea [63,68], and HRQL [25,61,62]. Resistance training also increases muscle mass and strength in sarcopenic COPD patients [15,70], and is also more effective than endurance training in counteracting muscle atrophy in COPD [71].

## 3. Endurance Training

Endurance training is defined by performing rhythmical contractions of low to moderate intensity (walking, running, cycling) for a relatively long period. Endurance training is often applied to improve aerobic capacity, which can be measured based on the maximal oxygen uptake (VO_2_ max) [41,72]. In healthy subjects, acute responses to endurance exercise include increased plasma volume, increased cardiac output, stroke volume, heart and breathing rate, and oxygen consumption [41,73]. When performed regularly, endurance training induces metabolic changes (increased net muscle protein balance, increased mitochondrial protein synthesis and mitochondrial density, enhanced triglyceride oxidation, attenuated muscle glycogen depletion) in the skeletal muscle, and cardiorespiratory system (decreased resting heart rate and blood pressure) [47,48,50,72,74,75]. These adaptations improve the delivery and utilization of oxygen for energy production and the capacity of prolonged exercise, thereby increasing the resistance to fatigue [41,72]. These adaptations arise from the activation of several regulatory pathways within the skeletal muscle, such as calcium-triggered regulatory pathways, the Akt-mTOR-P70S6K pathway, and AMPK, as well as MAPK signaling pathways [50,72,75].

### Endurance Training in Patients with COPD 

Endurance training has beneficial effects in patients with COPD. Previous studies investigating several endurance training programs in patients with COPD have shown significant improvements in 6MWD [25,60,76], peak oxygen uptake [25,61,63,77], endurance time or capacity [25,60,61,63,77], and HRQL [25,61]. In addition, endurance training has been shown to decrease dyspnea [63,77].

## 4. Neuromuscular Electrical Training

Neuromuscular electrical stimulation (NMES) is a training modality that can be applied during rehabilitation programs. It consists of electrical stimulation of the muscles through its motor nerve [78,79]. NMES is used to improve muscle strength, increase range of motion, and counteract muscle atrophy [78]. NMES can be modulated by stimulation frequency, pulse duration and intensity, contraction cycles, and training duration [78]. NMES induces action potentials in the muscles by depolarizing along the neuron to the sarcoplasmic reticulum (SR) in the motor end plate. Upon depolarization of the SR, calcium is released, allowing cross-bridge cycling that shortens and hereby contracts the skeletal muscle [79]. In healthy, active elderly patients, NMES training in addition to exercise training showed improvements in gait and balance [80]. Another study that investigated the effects of NMES training on endurance found that functional endurance is improved most in inactive patients with advanced disease when compared to active patients with stable symptoms [81].

### NMES in Patients with COPD

NMES is commonly used in rehabilitation programs of COPD patients with very severe dyspnea and lower-limb muscle weakness, as it evokes a relatively low demand on the respiratory system [82]. Thus, it enables training the muscles without experiencing severe dyspnea [83]. In these patients, high-frequency NMES (≥50 Hz) has been shown to improve quadricep muscle function [84,85], quadricep peak torque [83], 6MWD, endurance time, dyspnea, HRQL and ADL, exercise capacity, and health status [83,84,85]. A study conducted by Sillen et al. [83] showed that high-frequency NMES yields the same improvements in isokinetic quadricep muscle strength as resistance training in severely dyspneic patients with COPD.

## 5. Nutrition to Support Exercise Training Effects on Physical Functioning

Nutrition through meals and nutritional supplements enhance sport performance and promote adaptations to both endurance and resistance training [39]. As explained above, different modalities of exercise lead to different types of training adaptations on whole-body, cardiovascular, muscular, and cellular levels. The adaptive pathways that are activated by exercise are often also nutrient-sensitive, indicating that nutrition might not only play a direct role in acute exercise capacity but also in mediating longer-term adaptive responses to exercise [39,56,86]. Apart from nutrition per se, exercise capacity can also be enhanced by a variety nutritional supplements. An advantage of combining several nutritional supplements is that multiple pathways responsible for exercise capacity enhancement can be targeted. Some nutritional supplements can be applied to restore the energy balance, by increasing energy intake, while others can be used to specifically target pathways that may positively influence exercise training effects. The nutritional supplements that showed beneficial effects on exercise capacity when combined with exercise training in COPD are summarized in Table 1.

### 5.1. Nutritional Support 

It is widely known that carbohydrate (CHO) ingestion before and during endurance exercise plays a major role in improving different aspects of endurance performance [39,87,88,89]. However, a recent systematic review demonstrated that resistance training performance is not improved by both short- and long-term CHO supplementation in athletes, recreationally active individuals and untrained individuals [90]. This can be explained by the metabolic differences between resistance training and endurance training [90].

A recent meta-analysis that included healthy individuals reported that there is a dose-dependent association between the increase in muscle strength with resistance training and total protein intake up to 1.5 g/kg body weight per day [91]. Previously, a meta-analysis by Lin et al. [92] showed that protein supplementation during chronic endurance training further increased aerobic capacity, stimulated lean mass gain and improved time trial performance in healthy and clinical populations when compared to the control group. In addition, a review by McLellan et al. [93] concluded that adding protein to CHO supplementation does not have additional beneficial effects on endurance performance in healthy, active adults when CHO were delivered at optimal rates during and after exercise. Therefore, supplementing protein or CHO is an effective strategy to improve resistance or endurance performance, respectively, in healthy individuals.

### 5.2. Nutritional Support in COPD

A proportion of patients with COPD have increased resting energy expenditure and CHO oxidation when compared to healthy controls [32,94]. A recent meta-analysis showed that increased protein and/or energy intake improve anthropometric measures and handgrip strength in patients with COPD compared to controls [95]. When investigating the effects of a daily CHO-rich supplement of 570 kcal during PR, it was reported that shuttle walking performance improved in well-nourished and non-obese patients with COPD (BMI between 19 kg/m^2^ and 30 kg/m^2^) when compared to the control group [96].

A review by Kirk et al. concluded that in patients suffering from muscle-wasting disorders and chronic diseases, evidence is still conflicting with regards to beneficial effects of protein supplementation on muscle strength and physical function [97]. In patients with COPD, a study investigating the combination of protein and CHO supplementation and resistance training on lean body mass and strength after 8 weeks did not show additional effects of the supplementation as compared to resistance training alone [98]. However, acute supplementation of a meal rich in CHOs and protein had positive effects on maximal muscle strength after 24 h in patients with COPD when compared to no nutritional intervention [99]. While there are some studies that show no additional benefits from combining CHO and protein supplementation [93,98], other studies [96,99] suggest that a mix of CHOs and protein may be an effective strategy to increase energy intake and improve exercise capacity in patients with COPD when combined with exercise training.

### 5.3. Multinutrient Supplements

Multinutrient supplements are a way to combine several nutritional supplements at once to enhance exercise capacity through multiple pathways. A meta-analysis by Veronese et al. has shown that in elderly populations, multinutrient supplementation improved physical performance outcomes when compared with placebo [100].

In patients with COPD with low muscle mass (defined as FFMI under the sex- and age-specific 25th percentile FFMI values [101]), nutritional supplementation that included leucine, vitamin D, and omega-3 fatty acids for 4 months with supervised high-intensity exercise training showed additional improvements in lower-limb muscle strength and exercise capacity when compared to placebo [35]. In malnourished patients, nutritional drinks that consisted of CHO, protein, fats, omega-3 fatty acids, and vitamin A in combination with low-intensity exercise showed significant increases in body weight, FFM, quadricep muscle force, walking distance, and dyspnea when compared to a control group [34]. Another study conducted in muscle-wasted patients with COPD investigated liquid nutritional supplementation of 564 kcal per day for four months, combined with PR and reported increases in FFM, BMI, quadricep power, and exercise capacity when compared to usual care [102]. The results of these studies suggest that multinutrient, high-caloric supplements improve exercise capacity outcomes, but further research is required in larger populations.

### 5.4. Omega-3 Polyunsaturated Fatty Acids (PUFAs)

PUFAs are mainly found in oily fish and can influence skeletal muscle AA and insulin sensitivity, and thereby stimulate MPS through mTOR [43,103,104,105]. PUFAs also function as ligands for nuclear receptors such as peroxisome proliferator-activated receptors to upregulate genes that are involved in mitochondrial fatty acid metabolism [106]. In addition, PUFAs decrease inflammatory mediators and ROS production [105,107]. These characteristics of PUFAs may potentially lead to beneficial effects in exercise capacity. However, several reviews have shown inconclusive results as to whether PUFA supplementation improves exercise capacity in athletic populations [105,107,108].

A review by Wood et al. [109] reported that supplementing PUFAs without exercise training in patients with COPD was beneficial in reducing inflammatory responses, and improving muscle mass perseverance [109]. PUFA supplementation for 8 weeks during PR improved both incremental and constant work rate test performance, and muscle strength in COPD patients with a normal weight, to a greater extent than PR alone [110]. Based on the current evidence, further research needs to be conducted to explore the potential beneficial effects of PUFA supplementation in patients with COPD.

### 5.5. Caffeine 

Reductions in perceived effort, pain, and fatigue associated with exercise are the main effects of caffeine [39]. It can be used as ergogenic aid during elite endurance sports where small increases in performance can make a large difference [111]. According to a recent review and meta-analysis, acute caffeine supplementation improves maximal strength, power, muscular endurance, and movement velocity in healthy individuals [112,113]. In patients with COPD, acute caffeine supplementation did not improve incremental test performance and recovery after exercise when compared to placebo [114]. Future research should focus on larger populations to investigate the potential beneficial effects of acute and prolonged caffeine supplementation on exercise capacity in COPD.

### 5.6. Creatine 

Creatine is an AA-derived metabolite present in the skeletal muscle from dietary intake and endogenous synthesis in the liver [39,115]. In phosphorylated form, creatine can increase the phosphagen pool so that adenosine diphosphate can quickly be resynthesized into adenosine triphosphate in periods of high ATP turnover, as for example in sprints, weightlifting, and jumping events [115,116]. In addition, creatine can enhance high-energy phosphate diffusion between the mitochondria and myosin heads, which improves cross-bridge cycling and the maintenance of muscle tension. Lastly, creatine has a buffering function by using hydrogen ions formed during exercise for ADP re-phosphorylation, creating ATP [115]. Previous research has reported that activities that include repeated, short bouts of high-intensity exercise, such as soccer, football, and squash, would benefit most from creatine supplementation [115,117]. Creatine supplementation combined with resistance training has shown to have beneficial effects on muscle strength and muscle mass/fat free mass, as well as functional performance, when compared to control groups [118,119].

Previous research reported that muscle phosphocreatine (PCr) levels are lower in patients with COPD when compared to healthy subjects, and therefore might benefit from creatine supplementation [120]. However, a meta-analysis from 2010 concluded that creatine supplementation during PR did not show additional improvements in exercise capacity, muscle strength, and health-related quality of life, when compared to PR alone [121]. Therefore, it seems that creatine supplementation does not further enhance training effects when administered during PR.

### 5.7. Nitrate and Beetroot Juice (BRJ)

Dietary nitrate can mainly be derived from green leafy vegetables and plays an important role in the production of nitric oxide (NO) through the nitrate-nitrite-NO pathway [122]. NO can regulate skeletal muscle blood contractility, blood flow, calcium and glucose homeostasis, and mitochondrial biogenesis and respiration, hereby potentially influencing exercise performance [122,123]. In conditions of low oxygen availability and low pH, existing, for example, during exercise, nitrite can be reduced to NO in the blood and other tissues by a variety of enzymes and proteins, including deoxyhemoglobin [122]. Nitrate can be supplemented directly, but BRJ can also be used, as it is high in nitrate, cost-effective, and has the same potential in enhancing the nitrate-nitrite-NO pathway [123].

Previous research has shown that BRJ supplementation, beetroot consumption, or sodium nitrate supplementation decrease oxygen costs at the same power output [124,125,126] and improve time-trial performance in trained male cyclists [127,128] and recreationally fit adults [129], but have no ergogenic effects in highly trained endurance athletes [130,131,132,133]. With regard to resistance training, BRJ supplementation has shown improvements in power, total repetitions, muscular endurance, and velocity during resistance training when compared with placebo [123,134,135].

A meta-analysis investigating the beneficial effects of BRJ in patients with COPD, reported that BRJ supplementation decreased the Borg rating of perceived exertion (RPE) score, but had no beneficial effects on cardiovascular events, the 6MWD, cycling endurance time, and VO_2_max when compared to placebo [136]. However, two large, more recent studies conducted by Pavitt et al. [137,138] reported that BRJ supplementation in normal-weight patients with COPD showed improvements in exercise capacity when administered three hours before each training session during PR, and that it improved exercise endurance in hypoxic patients with COPD when compared to the placebo intervention. While the meta-analysis that concluded that BRJ did not have additional effects on exercise capacity included eight different RCTs (n = 243), the 2020 study conducted by Pavitt et al. [138] included 165 participants and showed improvements in endurance capacity in hypoxic COPD patients (n = 165), which might be an interesting subgroup of COPD that benefit from BRJ supplementation. However, further research should confirm these potential beneficial effects in hypoxic patients with COPD.

### 5.8. Beta-Alanine 

Beta-alanine, a non-essential AA, is the rate-limiting precursor for carnosine synthesis and buffering agent in the skeletal muscle [139]. In healthy elderly subjects, oral beta-alanine supplementation increases muscle carnosine content by 60–80% in 4–10 weeks without side effects [140,141,142,143]. Moreover, 4–12 weeks of daily oral beta-alanine supplementation without exercise training increased exercise capacity in untrained and aged individuals by 13–29% in comparison to placebo groups [141,144,145,146,147]. In cyclists, beta-alanine supplementation enhanced sprint performance at the end of an endurance training bout when compared with placebo [148]. However, beta-alanine supplementation did not seem to improve muscle recovery, whole-body strength, muscular endurance, and isokinetic force production after resistance training in untrained young adults when compared to resistance training alone [149,150].

Since patients with COPD suffer from reduced muscle carnosine stores and elevated exercise-induced muscle oxidative stress and acidosis, beta-alanine could potentially improve these conditions. Studies have shown that beta-alanine supplementation without exercise training in normal-weight patients with COPD significantly increased muscle carnosine levels up to 54% from baseline levels, but these results were not accompanied by improvements in exercise capacity, muscle oxidative stress, and quadricep function when compared to placebo, potentially due to the lack of an exercise training intervention [151,152,153]. Since beta-alanine supplementation can increase carnosine levels in the muscle, future research should focus on combining beta-alanine supplementation with exercise training to investigate whether this can translate to improved exercise capacity in patients with COPD.

### 5.9. Vitamin B12

Vitamin B12 is involved in metabolism, erythropoiesis, and immune function [154,155,156]. Potential beneficial effects on exercise capacity would arise from the vital role vitamin B12 plays in erythropoiesis [155]. However, vitamin B12 supplementation did not show improvements in exercise capacity, muscle strength, or endurance in adolescent boys and non-anemic men when compared to placebo intervention [157,158]. In patients with COPD, vitamin B12 supplementation in combination with exercise has not shown beneficial effects on exercise tolerance when compared to placebo [159]. However, further research in larger populations is needed.

### 5.10. Vitamin D

Vitamin D is an essential compound for bone mineralization, muscle growth, immune function, and cell differentiation [160]. Vitamin D2 is consumed from plant sources, and vitamin D3 is obtained via light exposure and dietary animal sources [160]. Previous research demonstrated that adequate vitamin D status is positively associated with endurance performance [161] and strength [162,163], but not with mean power production [164] and muscle recovery time [165].

Patients with COPD may have vitamin D deficiency [166]. In these patients, vitamin D plays an important role in combatting osteoporosis, promoting immune function and airway remodeling [154]. Higher vitamin D levels in patients with COPD have shown to be associated with improved pulmonary function [167]. A study investigating a monthly supplementation of vitamin D during PR showed larger improvements in VO_2_max when compared to placebo [168]. However, it has been shown that vitamin D3 supplementation does not enhance the effects of resistance training in older adults with or without COPD when compared to placebo [169]. Therefore, vitamin D supplementation may potentially effect endurance capacity, but further research is required.

### 5.11. L-Carnitine 

L-carnitine, an endogenous compound primarily synthesized in the liver and kidneys, and found predominantly in the skeletal muscle tissue, plays a great role in lipid metabolism by transporting long-chain fatty acids to the mitochondria [170,171,172]. Therefore, it is thought that L-carnitine can decrease weight through several mechanisms, such as reducing insulin resistance and increasing lipid oxidation [173]. A meta-analysis that investigated L-carnitine supplementation for weight management in overweight and obese adults showed that L-carnitine supplementation promotes a modest reduction in fat mass compared to placebo [171].

A study investigating the effects of L-carnitine supplementation in combination with a whole-body and respiratory muscle training program in patients with COPD, reported that L-carnitine did not significantly change body composition, but did enhance improvements in walking tolerance, heart rate, and blood lactate when compared to training alone [174]. Therefore, L-carnitine might be an interesting compound to improve exercise capacity, but further research in larger populations should be conducted to confirm these results.

**Table 1:** Overview of beneficial effects of combining exercise training and nutritional interventions to improve physical functioning in COPD.

Nutritional Intervention	Study	Supplement, Dose, Frequency	Exercise Intervention	Benefits from Combined Interventions
Multinutrient drinks and supplements	Steiner et al., 2003 [96] RCT N = 81	CHO-rich supplement, 570 kcal, 3 times per day. Trial duration: 7 weeks	14 PR sessions in 7 weeks, including endurance training and conditioning exercises	↑ shuttle walking performance in well-nourished patients
	Van de Bool et al., 2017 [35] RCT N = 81	Nutritional supplement with CHO, protein, fat, and enriched with leucine, vitamin D and PUFAs, 187.5 kcal per portion, 2–3 portions per day. Trial duration: 4 months	4-month outpatient PR program of 40 training sessions, including high-intensity endurance exercise, treadmill walking, and progressive resistance training	↑ lower-limb muscle strength ↑ cycle endurance time
	Sugawara et al., 2010 [34] RCT N = 32	Nutritional supplement with CHO, protein, and fat, enriched with PUFAs and vitamin A, 400 kcal, one time per day. Trial duration: 12 weeks	Home-based low-intensity exercise training, including upper and lower-limb exercises, level walking, and respiratory muscle training for 12 weeks in malnourished patients with COPD	↑ quadricep muscle force ↑ walking distance
	Huhn et al., 2022 [99] RCT N = 9	Acute supplementation of a meal rich in CHO and protein (white bun (60 g) with sour-milk cheese (100 g), one time before maximal strength tests. Trial duration: 2 times 2 days (crossover design)	A supervised strength training was performed by the patients according to usual protocol in physiotherapeutic setting. Maximal muscle strength was measured by knee extensor strength and chest press	↑ maximal muscle strength
	Van Wetering et al. 2010 [102] RCT N = 39	Liquid nutritional supplementation of 564 kcal per day. Trial duration: 4 months	Twice a week intensive supervised exercise training for 30 min	↑ muscle strength ↑ exercise capacity
PUFAs	Broek-huizen et al. 2005 [110] RCT N = 80	PUFA supplementation, 9 capsules of 1 g PUFA blend per day. Trial duration: 2 months	8-week PR program consisting of general physical training, including cycle ergometry, treadmill walking, swimming, sports, and games	↑ cycling performance ↑ muscle strength
	Van de Bool et al. 2017 [35] RCT N = 81	Nutritional supplement with CHO, protein, fat, and enriched with leucine, vitamin D and PUFAs, 187.5 kcal per portion, 2–3 portions per day. Trial duration: 4 months	4-month outpatient PR program of 40 training sessions, including high-intensity endurance exercise, treadmill walking, and progressive resistance training	↑ lower-limb muscle strength ↑ cycle endurance time
BRJ	Pavitt et al. 2020 [138] RCT N = 165	BRJ supplementation, 140 mL containing 0.8 g nitrate, consumed once, 3 h prior to exercise training during PR. Trial duration: 8 weeks	Patients were enrolled in PR that involved 8 weeks exercise training, including aerobic and strength training	↑ exercise capacity
L-carnitine	Borghi-Silva 2006 [174] RCT N = 16	L-carnitine supplementation, 1 g/day, two times a day. Trial duration: 6 weeks	6-week endurance training program of three one-hour training sessions per week. Each training session consisted of treadmill walking and inspiratory muscle training	↑ walking tolerance

## 6. Nutrition to Support Muscle Perseverance and Growth

The progressive decline in exercise capacity caused by skeletal muscle loss and dysfunction is one of the hallmarks of COPD [175,176,177]. Reduced muscle mass is associated with physical function impairment, frailty, and decreased HRQL, thereby adversely affecting mortality and morbidity [176,178,179]. Unintended weight loss due to increased energy requirements is common in patients with COPD and contributes to sarcopenia and frailty [19,180,181]. Therefore, dietary interventions that can prevent, diminish, or reverse the loss of muscle mass, and thus improve physical function, are of great clinical importance. Apart from nutritional interventions, there are also pharmacological treatments that could enhance muscle perseverance and growth. The beneficial effects of nutritional or pharmacological treatments combined with exercise training to target muscle maintenance and growth are summarized in Table 2.

### 6.1. Protein and EAA 

Adequate protein intake, in particular EAA, is crucial when following an exercise training program to enhance MPS, muscle buildup, and repair in health and disease [43,53,179,182,183,184]. Several studies have indicated that 20–25 g of high-quality protein, such as whey protein (corresponding to ~8–10 g EAA), maximally stimulates MPS in young/healthy adults [185,186]. A study that investigated the intake of 20 g of either whey protein, or mixed with casein (15 g whey + 5 g casein or 10 g whey + 10 g casein) in resistance-trained men showed no differences in body composition, muscle strength, or endurance, suggesting that whey and casein are equally effective protein sources [187]. However, in older adults, the elderly, or individuals with obesity or chronic diseases, the response to anabolic stimuli such as dietary protein intake and exercise is blunted [45,186,188,189,190,191,192,193,194]. This diminished response to the ingestion of dietary protein and exercise explains the difficulty of muscle maintenance in the elderly, but can be overcome by increasing the amount protein intake to optimize MPS, or by even combining protein or EAA feeding, and resistance exercise, which resulted in greater increases in MPS, than feeding on its own [191,195,196,197,198,199,200,201]. In addition, a review conducted by Kirk et al. [97] concluded that protein supplementation attenuates muscle loss in muscle-wasting disorders. Another study showed that high dietary protein intake in elderly was associated with less loss of muscle mass over time, hereby providing a strong rationale for adequate protein intake in sarcopenic elderly and sarcopenic COPD patients [202].

Protein quality and timing of intake are two important factors to optimize protein synthesis rates as well [43,203,204,205,206]. An even distribution of protein intake throughout the day (every 3–4 h) leads to higher protein synthesis rates and is associated with improved skeletal muscle mass and strength in older adults [207,208]. A meta-analysis investigating the effects of whey protein supplementation in overweight and obese individuals concluded that whey supplementation improves body weight and reduces total fat mass, along with some cardiovascular disease risk factors [209]. Currently, recommendations for the optimal amount of protein intake to manage sarcopenia in individuals with chronic diseases or who are malnourished varies between 1.2–2.0 g/kg body weight [177,210,211]. In patients with COPD, acute casein ingestion resulted in more protein anabolism when compared to whey protein during and following exercise [212]. Another study investigating EAA supplementation embedded in a 12-week PR program showed significant increases in bodyweight, and tended to increase in COPD patients with dynamic weight loss [183]. Therefore, protein supplementation may be an effective strategy to combat muscle mass loss in sarcopenic patients, and to stimulate muscle perseverance during weight loss in obese patients with COPD.

### 6.2. Leucine 

The essential AA leucine has three times the potency when compared to other EAAs in stimulating muscle anabolic signaling through the mTOR pathway [43,186]. It has been shown that leucine co-ingestion to casein protein prolonged the increase in myofibrillar protein synthesis rate after resistance training in older men [213]. In addition, another study that added leucine (3.4 g) to whey protein (16.6 g) reported no further increase in MPS when compared to 20 g whey protein intake [214]. Additionally, Moore et al. [185] did not find a difference in MPS when comparing 20 g of high-quality protein to 40 g of protein, suggesting that a saturating dose of 20–25 g of whey protein containing 2.5–3.0 g leucine optimally stimulates MPS [43].

In patients with COPD, several protein sources elicited comparable whole-body protein anabolic responses, with no additional effects observed when combined with leucine ingestion [215,216]. A study investigating a multinutrient supplement that also contained leucine showed that upon 4 months of supplementation combined with high-intensity exercise training, quadricep muscle strength, cycle endurance time and skeletal muscle mass improved in patients with COPD when compared with placebo [35]. The effects of leucine ingestion as single nutrient in combination with exercise training have not yet been established in patients with COPD, but might provide us with useful insights in the role of leucine in muscle maintenance and growth.

### 6.3. Anabolic-Androgenic Steroids (AAS) 

AASs are synthetic derivatives of the male hormone testosterone and have anabolic effects on the skeletal muscle through androgen receptor signaling [217]. In clinical practice, AASs are used to, for example, stimulate protein anabolism and treat male hypogonadism [218]. A meta-analysis conducted by Andrews et al. [219] concluded that AASs increase muscle strength and lean body mass.

In male patients with COPD, testosterone levels are lower when compared to age-matched control subjects with normal pulmonary function [220]. In addition, there has been shown that quadricep muscle weakness is related to low circulating levels in male COPD patients [220]. A recent meta-analysis has shown that AAS can improve body weight, FFM and peak workload, but not 6MWD and lung function when compared to the control group [221]. In addition, a study investigating treatment with nandrolone decanoate during PR showed higher increases in FFM when compared to placebo [222]. Another study investigating the administration of oral anabolic steroids in malnourished male patients with COPD for 27 weeks, in which the last five weeks included endurance training, showed increases in BMI, lean body mass, and anthropometrics, but no significant changes in endurance exercise capacity when compared to placebo [223]. The results of these studies suggest that AASs seem to improve muscle mass and anthropometrics in male patients with COPD, but further research should be conducted in larger populations to confirm whether these improvements are of clinical relevance.

### 6.4. Activin Type II Receptor Blockade 

The inhibiting growth factor myostatin acts predominantly through the activin type IIB receptor to negatively regulate skeletal muscle mass [175,224]. Inhibiting myostatin itself, or antagonizing its receptor activin type II, results in muscle growth in older sarcopenic adults and patients with COPD [190,225]. In overweight and obese adults with type 2 diabetes, the administration of Bimagrumab, an activin type II receptor antagonist, had positive effects on loss of fat mass and gains in lean mass when compared to placebo treatment [226].

Myostatin levels are elevated in sarcopenic and nonsarcopenic patients with COPD when compared to control groups, which are correlated to a reduced muscle mass [11,225,227,228,229]. A study investigating administration of Bimagrumab without exercise training in underweight patients with COPD showed an increase in muscle mass, but no improvements in functional capacity when compared to placebo [230]. This may suggest that increasing muscle mass via this mechanism is not enough to improve physical performance, or that potentially different factors of COPD may impact physical performance besides impacting muscle mass [179]. Future research should focus on the combination with exercise training and whether these beneficial effects are of clinical relevance.

### 6.5. Ghrelin 

Ghrelin is a hormone originally produced in the stomach that enhances hunger sensation and feeding, and could therefore potentially prevent weight loss in patients with COPD that cannot meet sufficient calorie intake due to reduced appetite [179,224,225]. In cachectic patients with COPD, ghrelin administration without exercise training improved body weight, lean body mass, muscle wasting, and exercise capacity [224,231,232,233,234]. Lastly, ghrelin administration combined with 3 weeks of PR showed only an additional reduction in symptoms when compared to placebo [234]. Therefore, ghrelin administration may possibly be an interesting option for cachectic patients with COPD with reduced appetite, to increase caloric intake and positively impact lean body mass and body weight.

**Table 2:** Overview of beneficial effects of combining exercise training and nutritional or pharmacological treatments to support muscle perseverance and growth.

Nutritional or Pharmaco-Logical Agent	Study	Supplement, Dose, Frequency	Exercise Intervention	Benefits from Combined Interventions
EAA	Baldi et al. 2010 [183] RCT N = 28	EAA supplementation, 4 g given twice a day. Trial duration: 12 weeks	Patients were enrolled in a PR program	↑ body weight ↑ FFM (*p* = 0.05)
Leucine	Van de Bool et al. 2017 [35] RCT N = 81	Nutritional supplement with CHO, protein, fat, and enriched with leucine, vitamin D and PUFAs, 187.5 kcal per portion, 2–3 portions per day. Trial duration: 4 months	4-month outpatient PR program of 40 training sessions including, high-intensity endurance exercise, treadmill walking, and progressive resistance training	↑ skeletal muscle mass
Anabolic-androgenic steroids (AAS)	Creutzberg et al. 2003 [222] RCT N = 63	Nandrolone decanoate administration, intramuscular injection of 50 mg on days 1, 15, 29 and 43 of PR. Trial duration: 8 weeks	All patients participated in an 8-week standardized PR program consisting of general physical training with particular attention to exercise in relation to daily activities such as cycle ergometry, treadmill walking, swimming, sports, and games	↑ FFM
	Ferreira et al. 1998 [223] RCT N = 23	Intramuscular injection of 250 mg testosterone at baseline, and 12 mg of oral stanozolol per day. Trial duration: 27 weeks	Patients enrolled in a PR program that included cycle ergometer exercises during weeks 18–27 of PR	↑ BMI ↑ lean body mass ↑ arm and thigh anthropometrics

## 7. Nutritional and Pharmacological Treatments to Support Weight Loss 

Obesity is a chronic disorder that is associated with increased risks of cardiovascular disease, metabolic syndrome, and all-cause mortality [36,235]. While previous research has paradoxically shown that mild–moderate obesity has protective effects on survival in patients with advanced COPD [236,237,238,239,240], studies show improved clinical outcomes upon weight loss [36,241]. Currently, there is little evidence available with regards to weight loss interventions in patients with COPD. However, considering the increasing prevalence of obesity in patients with COPD, nutritional strategies to reduce body weight and maintain muscle mass should be investigated.

### 7.1. Hypocaloric Diets

Hypocaloric diets focus on caloric restriction to target reductions in body weight, with the safe range for sarcopenic obese older adults being a calorie deficit between 200–700 calories per day [242,243]. Previous research has shown that energy restricted diets in obese older adults lead to the loss of fat mass but this is often accompanied by the loss of skeletal muscle mass [244,245,246]. Therefore, it is essential to combine high protein intake with a hypocaloric diet in obese patients to persevere muscle mass [241,242]. In obese patients with COPD, a low-calorie diet with matched protein intake based on bodyweight, in combination with resistance training showed significant improvements in BMI, exercise tolerance, health status, and muscle mass maintenance, and could therefore be appropriate approach to target weight/fat loss [36,241,242].

### 7.2. Glucagon-like Peptide-1 Receptor Agonists (GLP-1RAs)

GLP-1RAs reduce appetite and feelings of hunger, slow the food release from the stomach, and increase satiety [235,247]. Previous reviews have concluded that the use of GLP-1RA is an effective strategy to induce weight loss and decrease blood pressure in overweight and obese individuals [235,247]. Liraglutide, a GLP-1RA, is an approved pharmacological treatment for obesity [235].

Previous research conducted in obese patients with COPD has shown that liraglutide use without exercise training had significant beneficial effects on weight loss, forced vital capacity (FVC) and CAT-score, but not 6MWD when compared to the control group [248]. Apart from inducing weight loss, a recent review showed that the use of GLP-1RAs can reduce inflammatory responses, increase anti-inflammatory factors, and reduce oxidative stress damage, hereby improving lung function in patients with COPD [249]. The use of GLP-1RAs combined with exercise training has not yet been investigated in patients with COPD, but might provide us with useful insights with regards to weight management in COPD.

## 8. Novel Compounds 

In this section, several nutritional interventions will be discussed that have shown ergogenic potential in healthy populations, but of which there is little to no evidence available in COPD patients.

### 8.1. Sodium Bicarbonate 

Bicarbonate, the extracellular anion, manages electrolyte gradients and pH between intra and extracellular environments [250]. Endurance performance requires high rates of energy production from anaerobic glycolysis, which is often limited by the accumulation of hydrogen ions, therefore lowering the pH in the muscle and causing fatigue [39]. Upon ingestion of dietary bicarbonate, blood bicarbonate concentrations and blood pH rise, and can dispose these extra hydrogen ions that are produced from the working muscle through buffering [250]. A recent meta-analysis showed that sodium bicarbonate supplementation without exercise training acutely improves muscular endurance, but not muscular strength [251].

There is only one study that investigated acute sodium bicarbonate supplementation without exercise training in patients with COPD and a normal body weight, which showed no improvements in exercise tolerance when compared to a control group [252]. However, no study has investigated the combined effects of sodium bicarbonate and exercise training on exercise capacity.

### 8.2. Vitamins C and E

The effects of vitamins C and E have often been investigated together. Vitamin C, also known as ascorbic acid, has certain functions that may influence exercise capacity, such as producing carnitine, which transports long-chain fatty acids into the mitochondria; cortisol synthesis; and the uptake and transport of non-heme iron [154,155]. Vitamin E serves as an antioxidant of polyunsaturated fatty acid in subcellular structures and cell membranes, and influences the cellular response to oxidative stress [155]. Therefore, vitamin E may play a role in the oxidative metabolism of type I muscle fibers [155]. A recent review showed that supplementation with these vitamins did not improve exercise capacity outcomes in healthy individuals when combined with endurance training [253,254] as well as resistance training [255]. There were even indications that supplementation had negative effects on the adaptive responses to both endurance and resistance training [253,256,257,258,259]; however, this was not always confirmed [254,259,260].

Vitamin C and E supplementation without exercise training in patients with COPD has shown to be beneficial with regards to respiratory symptom improvements such as wheezing, phlegm production, and dyspnea [154]. However, to date, there have been no studies conducted investigating the effects of vitamin C and E supplementation as an adjunct to exercise training on exercise capacity in patients with COPD.

### 8.3. Resveratrol 

Resveratrol is a polyphenol compound derived from food, and may have antioxidant, anti-inflammatory, cardioprotective, and metabolic potential [261,262]. Resveratrol acts as an SIRT1 and AMPK activator, and therefore has the potential to promote mitochondrial metabolism and biogenesis in skeletal muscle [39,86,262,263,264]. A review conducted by Beijers et al. [261] found conflicting results with regards to the enhancement of mitochondrial metabolism by resveratrol. Furthermore, a study investigating 4-day oral resveratrol supplementation in male athletes did not show improvements in exercise capacity when compared with placebo [265]. Lastly, a study investigating daily supplementation of resveratrol combined with 8 weeks of high-intensity exercise training did not show metabolic improvements in healthy aged subjects when compared with placebo [266], and there are even indications that it may reduce the positive effects of exercise [266,267,268].

A recent study investigating the effects of resveratrol supplementation without exercise training in patients with COPD has shown no improvements in muscle mitochondrial biogenesis regulators AMPK, SIRT1, and PGC-1α; mitochondrial respiration; and inflammatory markers of adipose tissue compared with placebo treatment [269]. No studies have investigated the combined effects of resveratrol and exercise training in patients with COPD.

### 8.4. Magnesium 

Magnesium plays an important role in energy metabolism, protein synthesis, muscle contraction, and cell growth, and may therefore influence exercise performance [270,271]. However, a review from Zhang et al. [271] reported conflicting results with regards to ergogenic effects of magnesium to exercise training and further research is needed confirm potential beneficial effects.

Since a magnesium deficiency can trigger a low-grade inflammatory state, it could potentially worsen symptoms and pathophysiological mechanisms of COPD [270]. A recent study investigating the effects of magnesium supplementation in patients with COPD showed that magnesium potentially fulfils an anti-inflammatory role, but did not have beneficial effects on physical performance when compared with placebo [270]. The combination of exercise training and magnesium supplementation has not been investigated in patients with COPD.

### 8.5. (-)-Epicatechin 

(-)-Epicatechin is a cocoa-derived substance and is the active ingredient of dark chocolate, and has previously shown to enhance vascular health, skeletal muscle function, and fat oxidation [39,272,273,274,275,276,277]. A recent review has shown that (-)-epicatechin supplementation over several weeks is an efficient way to improve mitochondrial content and function in the muscle [278]. (-)-Epicatechin supplementation in normal and overweight adults showed indications that the supplement can promote mitochondrial biogenesis and enhance endurance-training adaptations in skeletal muscle [39]. However, another study showed no differences in anaerobic training adaptations, exercise capacity, and vasodilation upon (-)-epicatechin supplementation or dark chocolate consumption, and even showed indications of inhibiting aerobic and mitochondrial adaptations to cycling exercise [272,277,279]. One study has shown significant increases in muscle strength after combining an 8-week resistance training program with (-)-epicatechin in sarcopenic older males when compared to placebo [280]. Currently, there are no studies available that have investigated (-)-epicatechin supplementation in patients with COPD.

### 8.6. Nicotinamide Riboside (NR)

NR, a metabolite from vitamin B3, is a precursor for nicotinamide adenine dinucleotide (NAD^+^) synthesis in the skeletal muscle through the Nicotinamide Ribisode Kinase 1/2 (NRK1/2) pathway [39,281]. NAD+ has the ability to supply the mitochondrial electron transport chain by transferring electrons in its reduced form, NADH, to fuel oxidative phosphorylation in the mitochondria, producing ATP from adenosine diphosphate (ADP) [281]. NR is thought to influence skeletal muscle mitochondrial function through the NAD+/SIRT1/PGC-1α pathway [39]. A recent review investigating the effects of NR supplementation concluded that NR has few clinically relevant effects, with a reduction in inflammatory markers in the blood as main outcome [281]. Another review by Custodero et al. [282] concluded that human studies regarding NR supplementation and its effects on skeletal muscle and physical performance remain unclear. A study investigating the effects of acute NR supplementation on 5 k time trial performance showed that a single dose of NR does not enhance [283]. Currently, there are no studies available investigating the ergogenic effects of NR in patients with COPD.

### 8.7. β-Hydroxy β-Methylbutyrate (HMB) 

HMB, a leucine metabolite, potentially has anabolic properties in skeletal muscle [39,176]. Previous research has shown that HMB supplementation increased MPS through stimulating mTORC1 activity and attenuating MPB [284,285,286,287]. A recent meta-analysis by Phillips et al. [288] has reported that there is no evidence to support that HMB and supplements containing HMB, improve physical function in older persons or clinical populations.

One study conducted in patients with COPD on the intensive care unit, has shown decreased inflammatory and catabolic reactions upon HMB supplementation [289]. However, no studies have been conducted that investigate the effects of HMB in combination with exercise training in ambulant patients with COPD. Further research should focus on the potential additional effects of HMB supplementation combined with exercise training in patients with COPD.

## 9. Summary

**Figure 1:**
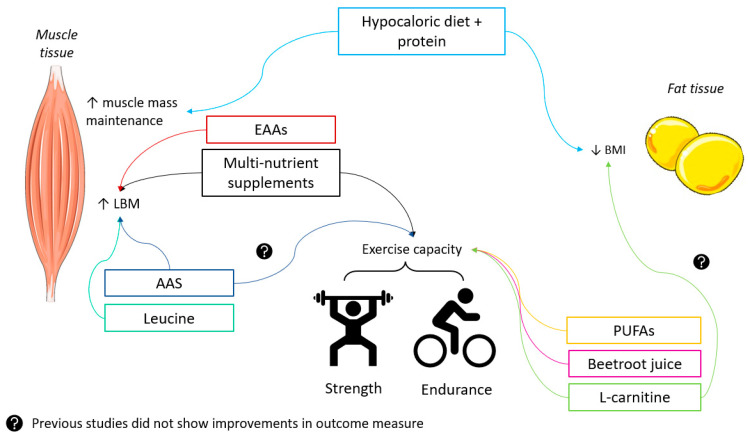
Overview of all nutritional and pharmacological treatments combined with exercise training and their effects on exercise capacity, muscle mass, maintenance and growth, and weight loss. Abbreviations: EAAs: essential amino acids; AAS: anabolic androgenic steroids; PUFAs: polyunsaturated fatty acids; LBM: lean body mass; BMI: body mass index [34,35,96,99,102,110,138,174,183,223,224,243].

In this review, the effects of combinations of exercise and nutritional or pharmacological treatments to target the different metabolic phenotypes of COPD were described. Apart from the implication of a well-balanced diet during PR in patients with COPD, this review describes some nutritional interventions and pharmacological treatments in combination with exercise training that could be beneficial for patients with COPD. In addition, considering the relation between COPD and lung cancer, and in light of prehabilitation, some of the described interventions in this review may also potentially be beneficial for lung cancer patients with underlying COPD. A summary of these potential beneficial effects is presented in Figure 1.

With respect to enhancing exercise training effects, patients with COPD may potentially benefit from supplements such as PUFAs, BRJ, L-carnitine, protein, or EAA to improve muscle strength and exercise capacity, but further research should be conducted to confirm these beneficial effects. Vitamin D and vitamin B12 did not show beneficial effects on exercise capacity on their own, or when combined with exercise training. Caffeine, creatine, sodium bicarbonate, beta-alanine, vitamin C, vitamin E, resveratrol, and magnesium did not show beneficial effects on exercise capacity upon supplementation, and future research should focus on the potential additional effects of combining these supplements with exercise training in COPD.

To target the sarcopenic and cachectic metabolic phenotypes, combinations of nutritional and pharmacological treatments and exercise training aiming to increase muscle mass are feasible. While gaining muscle mass is predominantly important for the sarcopenic and cachectic phenotypes, maintaining muscle mass is relevant for all metabolic phenotypes of COPD and can be achieved with adequate protein, EAA, and leucine intake together with resistance training. The use of AAS has shown to increase muscle mass in male patients with COPD, and the use of Bimagrumab has shown to increase muscle mass in underweight patients with COPD. These pharmacological treatments might potentially be an interesting approach for the sarcopenic phenotypes of COPD, especially when combined with exercise training, but future research is required to investigate this. Lastly, ghrelin has shown to improve body weight and lean body mass in cachectic patients with COPD. Future research should focus on including larger populations and subpopulations such as sarcopenic patients with COPD.

To target the obese metabolic phenotype, nutritional and pharmacological approaches potentially combined with exercise training can be applied to enhance fat loss. Hypocaloric diets in combination with matched protein intake and resistance training have shown the most promising positive results on improving and maintaining muscle mass, and reducing fat mass in obese patients with COPD. Furthermore, liraglutide has shown interesting results in obese patients with COPD with regards to weight loss, but further research should be conducted including the obese metabolic phenotypes of COPD. Lastly, L-carnitine supplementation has shown promising effects on weight management in overweight and obese adults, but future studies should focus on investigating these effects in obese and sarcopenic obese patients with COPD.

Taking away from the current review, tailoring exercise training, nutritional and pharmacological agents to patient characteristics could probably maximize the effects of PR.

## Data Availability

Data are contained within the article.
